# Genetic Variability and Molecular Evolution of Tomato Mosaic Virus Populations in Three Northern China Provinces

**DOI:** 10.3390/v15071617

**Published:** 2023-07-24

**Authors:** Jinfu Lyu, Yuanyuan Yang, Xiaohui Sun, Shanshan Jiang, Hao Hong, Xiaoping Zhu, Yongguang Liu

**Affiliations:** 1 Shandong Provincial University Laboratory for Protected Horticulture, Shandong Facility Horticulture Bioengineering Research Center, Weifang University of Science and Technology, Shouguang 262700, China; 2 Shandong Province Key Laboratory of Plant Virology, Institute of Plant Protection, Shandong Academy of Agricultural Sciences, Jinan 250100, China; 3 Collaborative Innovation Center of Fruit & Vegetable Quality and Efficient Production, College of Plant Protection, Shandong Agricultural University, Tai’an 271018, China

**Keywords:** phylogenetic analysis, tomato mosaic virus, genetic diversity, ToMV strain variation

## Abstract

RNA viruses tend to mutate during transmission and host infection, which is critical to viral adaptation and evolution. Tomato mosaic virus (ToMV) is a member of the genus *Tobamovirus* (family *Virgaviridae*) and an economically important virus with detrimental effects on tomatoes worldwide. Although the ToMV gene sequences have been completed in China, their genetic diversity and population structure remain unclear. We collected 425 tomato samples from tomato-growing areas in three northern Chinese provinces 2016. Reverse transcription PCR results showed that the average incidence of the virus in the field samples was 67.15%, and ToMV was detected in all test areas. The analysis of ToMV single nucleotide polymorphisms in China showed that ToMV was evolutionarily conserved, and the variation in the whole genome was uneven. Pairwise identity analysis showed significant variability in genome sequences among ToMV strains with genomic nucleotide identities of 73.2–99.6%. The ToMV population in the northern Chinese provinces had purification and selection functions, which were beneficial in the evolution of the ToMV population. Although there has been some distribution of ToMV strains in China, the virus was generally stabilized as a uniform strain under the pressure of purification selection. Our findings show how to monitor the prevalent strains of ToMV and their virulence in China and provide useful information for its prevention and control.

## 1. Introduction

Tomato (*Solanum lycopersicum*) is a popular fruit and vegetable of economic importance [1], and China is the world’s largest tomato-producing country [2]. However, viral diseases have seriously affected tomato yield and quality [3,4,5,6,7], limiting global tomato trade [8]. The tomato mosaic virus (ToMV), a member of the genus *Tobamovirus* (family *Virgaviridae*), is one of the most prevalent and devastating viruses affecting tomato production [9].

ToMV is a common viral pathogen [10] that was first described in 1909 and reported specifically in China in 1993 [11,12]. ToMV can spread rapidly and affect all susceptible tomato varieties, with symptoms including leaf discoloration and shrinkage, vein clearing, a mottling or mosaic-like leaf appearance, necrosis, stunted plant growth, and flower dropping, especially during fall in open field tomatoes, with a disease incidence ranging from 10–100% and causing approximately 20% of the total losses in tomato production globally [13]. The damaging effects are not confined to tomatoes, as ToMV has a wide host range [7,14].

Tobamoviruses cause severe damage to solanaceous vegetables [7] and can be divided into three subgroups according to their host range, genome structure, and evolutionary relationship [15]. Subgroups I, II, and III mainly infect Solanaceae, Cucurbitaceae, Leguminosae, and Brassicaceae, with, ToMV belonging to subgroup I of tobamoviruses [16].

The 3′ untranslated region (UTR) of the ToMV genome contains tRNA-like sequences that bind histidine specifically in vitro [17], while the 5′ UTR has a 71-nucleotide noncoding sequence in its cap structure that is rich in adenylate and uridylate and regulates the expression of the 5′ UTR. The genome contains four open reading frames (ORFs), where ORF 1 and 2 encode the 126 and 183 kDa replication proteins, ORF3 encodes the 30 kDa movement protein (MP) that is essential for the intercellular movement of viruses, and ORF 4 encodes the 17.5 kDa coat protein (CP), which is mainly involved in the long-distance transport of viruses. MP and CP are generated by the translation of different subgenomes during viral infection [17].

With each infection, epidemic, and spread of the ToMV virus, the virus’s genetic material changes [18]. In this study, the entire ToMV genome was determined and analyzed to compare the differences between its occurrence in China and abroad and to clarify its occurrence and transmission properties, which are significant in preventing and controlling viral damage and spread. This study examines the status and evolution trend of the ToMV variation and the evolution trend, which has broad application to both research and field growth programs.

## 2. Materials and Methods

### 2.1. Sample Collection and Virus Detection

A total of 425 tomato plants showing symptoms of viral infection were collected from commercial greenhouses cultivating tomato crops from seven locations in Shandong Province (Taian, Weifang, Liaocheng, Zibo, Jining, Yantai, and Dezhou), one in Shanxi Province (Jinzhong) and one from Inner Mongolia (Hohhot) in 2016. Foliar symptoms included a mosaic-like discoloration, mottling, yellowing, malformation, necrosis, narrowing, and rolling. Brown necrosis was observed on the stems, pedicles, and sepals. Affected tomato fruits displayed deformities which reduced the commercial value of tomatoes. A typical symptom was brown necrotic lesions on fruits. Yellow spots were also found on the fruits of some tomato cultivars.

Total RNA was extracted from a 0.1 g sample of fresh leaves collected from symptomatic and asymptomatic tomato plants using an RNA Simple Total RNA Purification Kit (TIANGEN Biotech, Beijing, China). First-strand cDNA synthesis was performed using reverse transcriptase M-MLV RNase H- (TaKaRa, Dalian, China) in a 10 μL reaction mixture according to the manufacturer’s instructions. The specific ToMV-f/ToMV-r primers (Table 1) amplifying the complete coat protein (CP) sequence were used to detect ToMV from cDNA samples. PCR amplification was performed using EasyTaq DNA polymerase (Trans, Beijing, China). PCR was performed in 25 μL reaction mixtures containing 2.5 μL Easy Buffer, 0.25 mM of each dNTP, 1 μM of each primer, 2 μL cDNA, 0.5 μL EasyTaq polymerase (5 U/μL), and ddH_2_O water to 25 μL. The PCR program conditions were as follows: initial denaturation at 94 °C for 5 min, followed by 40 cycles at 95 °C for 40 s, 51 °C for 45 s, and 72 °C for 1 min; and a final elongation step at 72 °C for 10 min. Each PCR product was electrophoresed in 0.8% agarose gel, stained with ethidium bromide, and visualized using a UV transilluminator. The products were purified and cloned into pMD18-T vectors (TaKaRa, Dalian, China). Twenty-six ToMV whole genome sequences and seven ToMV CP sequences were determined and deposited in GenBank.

### 2.2. Whole Genome Amplification, Sequence Alignment, and Phylogenetic Analysis

Three primers were designed to amplify the entire genome (Table 1). The 5′ and 3′ terminal regions were amplified using the SMART RACE cDNA Amplification Kit and the 3′-FULL RACE Core Set (TaKaRa) following the manufacturer’s instructions. For amplification of the 5′-end sequence, first-strand cDNA was synthesized from viral genomic RNA using TO5SP1 primers, and a poly(A) tail was added to the 3′-end of cDNA with terminal transferase. PCR was then performed using the TO5SP2 and oligo-dT-anchor primers (5′-GACCACGCGTATCGATGTCGACTTTTTTTTTTTTTTTTTV-3′). For amplification of the 3′-end sequence, total RNA was denatured in hot water and first-strand cDNA was synthesized using primers TO3SP2 and poly (A), as described. PCR was performed using TOP3SP1 and oligo-dT anchor primers. The products obtained were then cloned and sequenced as previously described. The primer sequences used for RACE are listed in Table 1. The sequence was read using Chromas software (Chromas version 2.6.5, Technelysium Pty Ltd.), and the peak value was observed. The sequence was edited and corrected using the DNAStar software package. The processed sequences were analyzed using the NCBI sequence alignment tool BLAST (https://blast.ncbi.nlm.nih.gov/ (accessed on 26 February 2018). DNAStar software (DNAStar Inc., Madison, WI, USA) was used to analyze nucleotide similarities. A phylogenetic tree was constructed using the maximum-likelihood algorithm using MEGA7 software [18]. The tobacco mosaic virus was included as an outgroup. The amplified sequences in this study and the ToMV sequences from the NCBI database were used to construct the phylogenetic tree (Table S1).

### 2.3. Estimation of Genetic Diversity Parameters and Population Differentiation

To analyze gene exchange and epidemic trends of ToMV in several endemic areas, the ToMV sequences of vegetable growing areas with contiguous facilities were divided into four regions: A (Asia), B (Europe), C (Africa), and D (America). The number of variable sites (S), nucleotide diversity (h), and haplotype diversity (Hd) were used to analyze the sequences of these four regions. To assess the selection pressure in each ToMV coding region, the ratio of non-synonymous (dN) to synonymous (dS) substitution rates (ω = dN/dS) was calculated using MEGA7 software [19]. |Fst| Statistics and gene flow among six provinces of China (Shandong, Shanxi, Inner Mongolia, Beijing, Zhejiang, Gansu) using DNASP v6 software [20].

### 2.4. Recombination Analysis

To analyze ToMV gene exchange and epidemic trends in several disease-occurrence areas in China, ToMV genetic diversity analysis was performed using DNASP v6 software [20]. The recombination detection program version 4 (RDP4) [21] was used to detect and analyze recombination. Evidence of recombination was further analyzed using seven algorithms (RDP, GENECONV, BootScan, Maximum Chisquare, Chimaera, SisScan, and 3Seq) in RDP4 with a Bonferroni corrected *p*-value cutoff of 0.05 [21]. A putative recombination event was considered significant if it was supported by at least four of the seven different methods, with an associated *p*-value < 1 × 10^−6^ [21].

## 3. Results

### 3.1. Field Symptoms and ToMV PCR Identification

**Figure 1:**
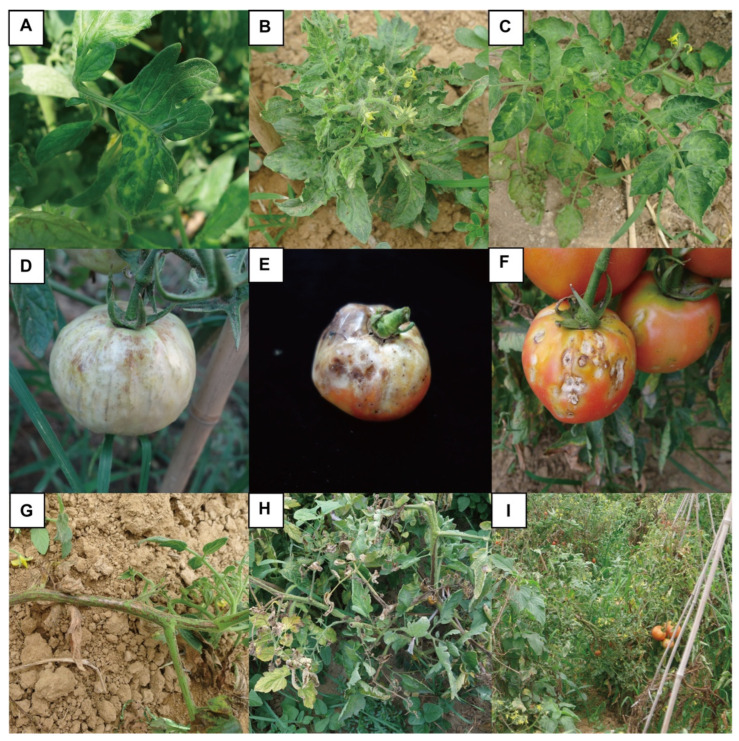
The tomato mosaic virus (ToMV) infection symptoms occur in tomato leaves, fruits, and stems. (**A**–**C**) Symptoms of ToMV in tomato leaves. (**D**–**F**) Symptoms of ToMV in tomato fruits. (**G**–**I**) Symptoms of ToMV in tomato pedicles stems, and sepals.

The ToMV-induced symptoms observed in tomato plants in the field were complex. Foliar symptoms included a mosaic-like leaf appearance and leaf mottling, yellowing, malformation, necrosis, narrowing, and rolling (Figure 1A–C). Affected tomato fruits displayed deformities. A typical symptom was brown necrotic lesions on fruits. Yellow spots were also found on the fruits of some tomato cultivars (Figure 1D–F). Brown necrosis was observed on the pedicles, stems, and sepals (Figure 1G–I). The viral RNAs from typically symptomatic tomato samples were amplified by an approximately 670 bp fragment, and the occurrence of ToMV was confirmed by sequencing. Seven ToMV CP sequences were determined and deposited in GenBank (Table S1). The detection rates are shown in Table 2. The complete genome was amplified from the sample by PCR amplification with three pairs (ToMV-1F/ToMV-1R, ToMV-2F/ToMV-2R, and ToMV-3F/ToMV-3R) combined with rapid amplification of cDNA ends (RACE) primer pairs (TO5SP1, TO5SP2, TO3SP1, and TO3SP2) (Table 1). In 2016, 425 samples were collected in the facility production areas, and representative positive samples were cloned. A total of 26 full-length ToMV sequences were cloned (Table S1).

### 3.2. Phylogenetic Relationship among ToMV Strains

**Figure 2:**
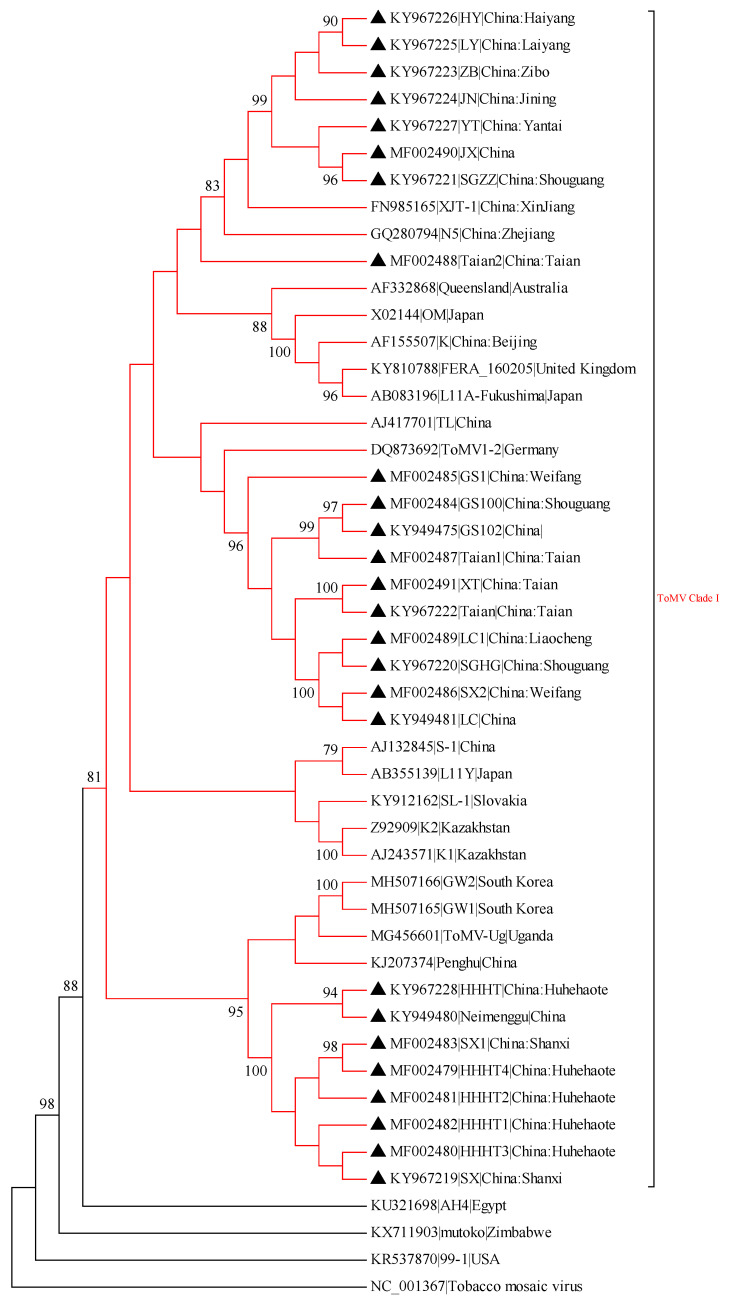
Maximum-likelihood (ML) phylogenetic tree constructed with MEGA7 using the complete genome sequence of tomato mosaic virus. The ML analysis was conducted using the nucleotide identity distances and 1000 bootstrap replicates (bootstrap values > 70% are shown at the nodes). The virus strains are highlighted by black triangles. An isolate of the tobacco mosaic virus was used as an outgroup.

**Figure 3:**
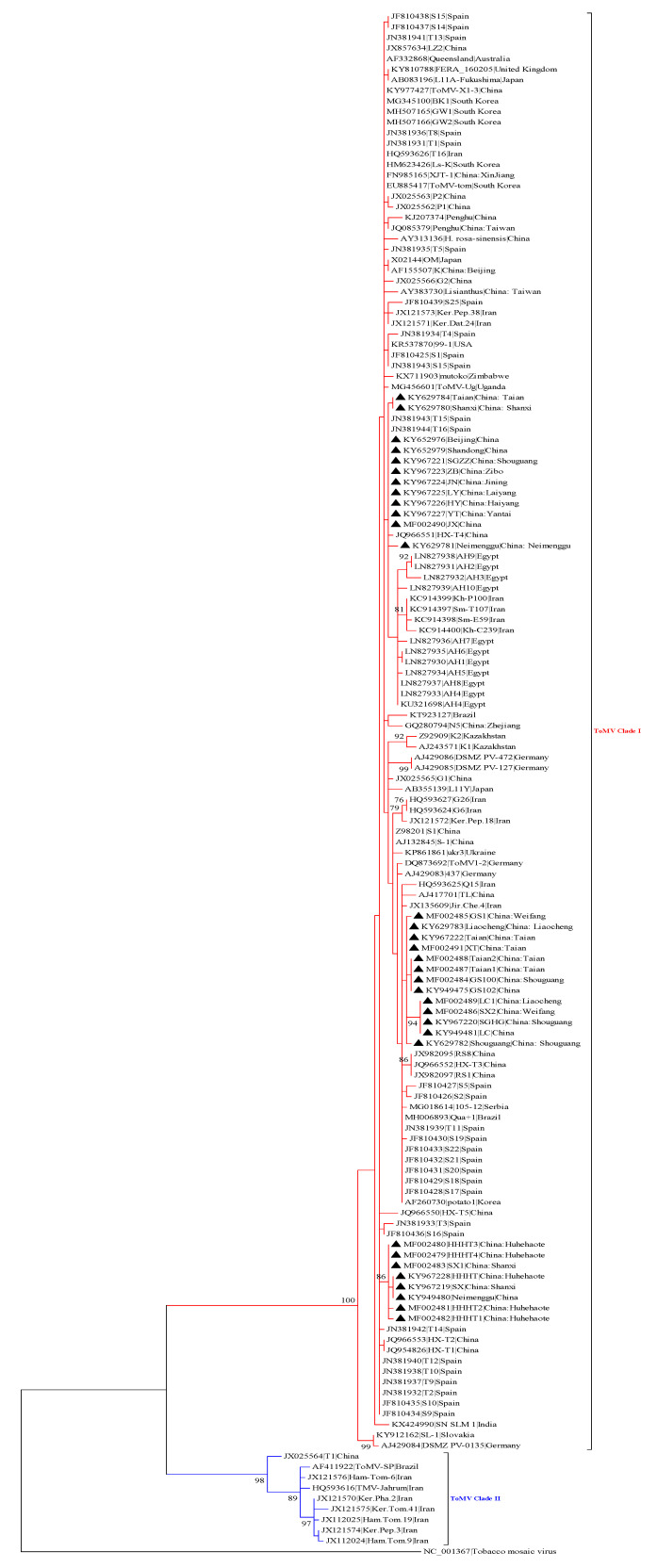
The maximum-likelihood (ML) phylogenetic tree was constructed using MEGA7 based on the coat protein sequence of the tomato mosaic virus (ToMV). The analysis was conducted using nucleotide identity distances and 1000 bootstrap replicates (bootstrap values > 70% are shown at the nodes). The virus strains are highlighted by black triangles. An isolate of the tobacco mosaic virus was used as an outgroup.

A phylogram was generated from the alignment of nucleotide sequences of full-length ToMV isolates and isolates from China, Japan, South Korea, Kazakhstan, Slovakia, Australia, USA, Germany, United Kingdom, Egypt, Zimbabwe, and Uganda (Table S1). The evolutionary tree demonstrated that all ToMV strains could be grouped into two main clades. The Clade I virus strains were mainly from China, Kazakhstan, Japan, Australia, Egypt, and other countries. The Shandong isolates (SGHG, LC, SX2, LC1, XT, Taian, GS1, GS100, GS102, Taian1, Taian2) are grouped into -Clade I and -separate branch. Seven isolates (JX, SGZZ, JN, HY, LY, ZB, YT) with Xinjiang (XJT-1) and Zhejiang (N5) isolates grouped into a small clade that was closely related. Inner Mongolia isolates (Neimenggu, HHHT, HHHT1, HHHT2, HHHT3, HHHT4), Shanxi isolates (SX, SX1) were closest to Uganda (ToMV-Ug), South Korea (GW1, GW2), and China Penghu) isolates. All ToMV genome-wide isolates amplified in this study belong to Clade I (Figure 2). The evolutionary tree based on ToMV coat protein shows that all ToMV isolates fall into three categories. Similar to genome-wide evolutionary analysis, the ToMV CP isolates amplified in this study were all located in Clade I (Figure 3).

### 3.3. Homology and Variation among ToMV Strains

The genome sequences obtained from parts of the three northern provinces were compared for nucleotide identity. ToMV sequences (SGHG, LC, SX2, LC1, XT, Taian, GS1, GS100, GS102, Taian1, Taian2, JX, SGZZ, JN, HY, LY, ZB, YT, Neimenggu, HHHT, HHHT1, HHHT2, HHHT3, HHHT4, SX, SX1) were compared with ToMV sequences from different regions in China and abroad. The comparison results showed that Shandong strains (SGHG, LC, SX2, LC1, XT, Taian, GS1, GS100, GS102, Taian1, Taian2, JX, SGZZ, JN, HY, LY, ZB, YT) had the highest consistency with the isolates of Xinjiang (XJT-1) and Zhejiang (N5). Inner Mongolia strains (HHHT, HHHT1, HHHT2, HHHT3, HHHT4) and Shanxi strains (SX, SX1) had the highest consistency with Uganda (ToMV-Ug), South Korea (GW1, GW2), and China (Penghu) isolates (Table S3).

The analysis showed that compared to the 25 sequences obtained in this study and 21 GenBank sequences, the 5′ UTR of the GS100 isolate had a nucleotide consistency of between 97.3 and 100%. The 184 kDa protein showed a 96.7–99.9% nucleotide consistency, and the 126 kDa protein showed 98.7–99.8%. This study’s consistency between the MP protein and 46 sequences was 97.5–100%. Among the CP protein and the 46 nucleotide sequences obtained in this study, the consistency was 97.7–100%. The consistency between the 3′-UTR and the 46 nucleotide sequences was 93.2–100.0%. The nucleotide consistency ranged from 73.2–99.6% with other ToMV members (Table S2).

The 5′ UTR, 126 kDa, 183 kDa, MP, CP, and 3′ UTR mutation rates were compared with the representative whole genome sequence (ToMV 1-2) from Germany. The 5′ UTR consisted of 71 nucleotides, among which four nucleotides were mutated, and the mutation rate was 5.6%. The 126 kDa protein gene consisted of 3351 nucleotides, which translated to 1117 amino acids, of which 64 nucleotides and seven amino acids were mutated with mutation rates of 1.9% and 0.6%, respectively. The 183 kDa protein gene comprised 4851 nucleotides translated to 1617 amino acids, among which 93 nucleotides and 13 amino acids were mutated with mutation rates of 1.9% and 0.8%, respectively. The MP gene was composed of 795 nucleotides, which translated to 265 amino acids, among which 12 nucleotides were mutated with a mutation rate of 1.5% and an amino acid mutation rate of zero. The CP gene consisted of 480 nucleotides, which translated to 160 amino acids, of which 12 nucleotides and 1 amino acid were mutated with mutation rates of 2.5% and 0.6%, respectively. The 3′ UTR consisted of 201 nucleotides, of which six were mutated with a mutation rate of 2.9%. Mutation information is presented in Table 3.

### 3.4. Estimation of Genetic Diversity Parameters and Population Differentiation

Estimation of genetic diversity parameters and population differentiation are shown in Table 4. The nucleotide diversity of the four regions was in the range of 0.00807–0.07951. The haplotype diversity value was approximately one. These results showed that the nucleotide and haplotype diversity of the ToMV population in the four regions did not show high nucleotide diversity. The nucleotide polymorphism analysis of the four regions showed that the values of Tajima’s D, Fu’s D, and Li’s F were negative but significant. The dN/dS values of Asia, Europe, Africa, and America were 0.0662, 0.138, 0.135, and 0.0553, respectively, indicating that the ToMV populations in the four regions had the function of purification and selection. The non-synonymous mutations were more prevalent than the synonymous mutations, further indicating that the population was in purifying selection, which was beneficial to the evolution of the ToMV population. Figure S1 shows that the population change curve in the four regions presents a zigzag and uneven change state, indicating that the population was in equilibrium in the four regions. The genome-wide gene flow of ToMV was analyzed using DNASP version 6. The study of gene exchange level usually uses the absolute value of the Fst coefficient (|Fst|). An |Fst| value ranges from 0 to 1, with an|Fst| > 0.25 indicating that the two population was seriously differentiated. In the genetic analysis of ToMV populations in different regions, the genetic distance between America and Africa was represented by an |Fst| of 0.07137. Between America and Europe, the |Fst| was 0.00734, between Asia and America, it was 0.09459, between Africa and Europe, it was 0.38170, between Africa and Asia, it was 0.13702, and between Europe and Asia, the |Fst| value was 0.04543 (Table S4). Through analyzing ToMV in gene exchange between different regions, we found that in addition to Africa and Europe having an |Fst| greater than 0.25, showing that ToMV communication between Africa and Europe was seriously differentiated. Between the other regions, gene exchange is frequent. The lowest |Fst| value was calculated between America and Europe, indicating that the most frequent sharing of ToMV genetic material occurred between America and Europe.

In the genetic analysis of ToMV populations in six Chinese provinces, the genetic distance between Shandong and Shanxi was represented by an |Fst| of 0.34000. Between Shandong and Inner Mongolia, the |Fst| was 0.50413, represented by an |Fst| of 0.34000. Between Shandong and Inner Mongolia, the |Fst| was 0.50413. Between Shanxi and Zhejiang, it was 0.35294. Between Inner Mongolia and Zhejiang, it was 0.51145. Between Shanxi and Zhejiang, it was 0.35294. Between Inner Mongolia and Zhejiang, it was 0.51145; and between Inner Mongolia and Gansu, the |Fst| value was 0.35632 (Table S5). |Fst| greater than 0.25 between these regions, showing that populations of ToMV between these regions were seriously differentiated, and between the other regions, gene exchange is frequent. The lowest |Fst| value was calculated between Shanxi and Beijing, indicating that the most frequent sharing of ToMV genetic mate-rial occurred between Shanxi and Beijing (Table S5).

### 3.5. The ToMV Genetic Organisation Is Highly Conserved

**Figure 4:**
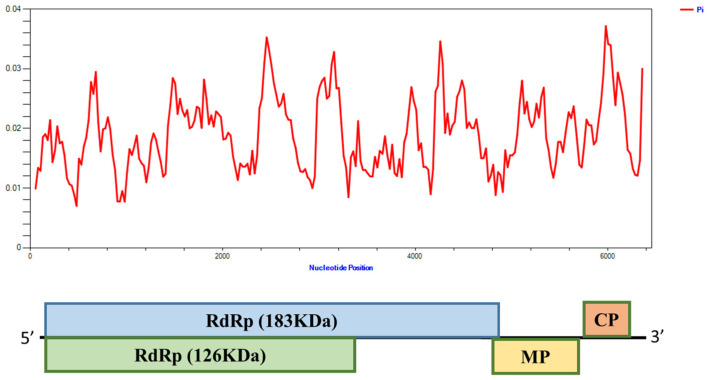
Distribution of nucleotide diversity along 47 tomato mosaic virus (ToMV) whole-genome sequences. The nucleotide diversity (*Y*-axis) was plotted against nucleotide position (*X*-axis) using DNASP v6 with a 200-nucleotide (nt) sliding window and a 25-nt step size. MP = movement protein, CP = coat protein.

The results of the ToMV single nucleotide polymorphism analysis are shown in Figure 4. ToMV was more conserved in evolution, and the variation in the whole genome was uneven. The maximum value of the maximum variation peak was at the overlap of the ORF of the 183 kDa and 126 kDa protein genes, while the 30 kDa and 17.5 kDa protein genes showed relatively little change between ToMV isolates. Analysis of ToMV CP mutations in 6 provinces in China showed that there were 16 base mutations in the CP sequence in the Shandong region, among which one amino acid had double base mutations, ten synonymous mutations and five non-synonymous mutations. There were six base mutations in the CP sequence in the Shanxi region, including three synonym mutations and three non-synonym mutations. There are five base mutations in the CP sequence in Zhejiang province, including four synonymous and one non-synonymous mutations. There were eight base mutations in the CP sequence in the Beijing area, including seven synonymous mutations and one non-synonymous mutation. There are 13 base mutations in the CP sequence in the Gansu region, containing nine synonymous mutations and four non-synonymous mutations; There are nine base mutations in the CP sequence in Inner Mongolia, including four synonymous mutations and five non-synonymous mutations. Base variation characteristics of CP contribute to adaptation to the host or environment.

### 3.6. Recombination Analyses

Recombination is the leading factor in the evolution and emergence of the virus; therefore, we performed a recombination analysis using RDP4. We did not find any evidence of recombination in the nucleotide sequences of the newly identified ToMV strains.

## 4. Discussion

ToMV was first reported in 1909 in the USA and can infect a wide range of plant species, causing losses in many economically important crops [12]. ToMV is widely distributed in many countries and global regions, including China [10,16,22,23,24,25]. For the past several years, the presence of ToMV in tomato growing areas has been dominant and consistent in three provinces of China due to the stable nature of its viral particles, its long survival period in crop hosts, its easy mechanical and rapid transmission through field workers and agricultural instruments [26], and its resistance to elimination. However, ToMV can have various strains with different virulence, which makes exploring and understanding the diversity, occurrence, and distribution of all ToMV strains important for successful breeding and evaluation of disease resistance of crop plants [27]. Our findings indicated that the ToMV strains collected from multiple locations in China were relatively uniform, stable, and conservative, which could have been associated with the high virulence of the current and prevalent ToMV strain or the selection pressure of a uniform, weak, or horizontal resistant gene in the tomato population.

In this study, ToMV was detected at all locations sampled. Its detection rate in Hohhot, Shanxi (Jinzhong) and Shandong (Shouguang, Yantai, Taian, Dezhou, Liaocheng, Jining, and Zibo) in 2016 was between 16.67% and 100%, indicating that ToMV was quite common in these areas and needed serious attention from local tomato growers and government agencies to control and monitor its infection rates. To safeguard agriculture and diagnostic security against new isolates or potential variants that can threaten solanaceous commodities, we characterized the genetic diversity of ToMV isolates introduced into China and worldwide. Several ToMV isolates were collected from leaves, fruits, and stems at commercial greenhouses cultivating tomato crops in three northern Chinese provinces. We also analyzed the genetic composition of the genomes and conducted various genetic analyses to understand the drivers of ToMV diversity. Based on the results of this study, we found that ToMV has very limited genetic diversity across genomes. Furthermore, ToMV diverges from the neutral evolutionary theory, which indicates the virus is not undergoing natural selection and that accumulated mutations across the genomes are low-frequency and random. Furthermore, this divergence from neutrality is most likely caused by a population expansion of ToMV, a high number of haplotypes across different genes, and low nucleotide diversity.

In this study, ToMV was more conserved in evolution, and the variation in the whole genome was uneven. The maximum value of the variation peak was at the overlap of the ORF of the 183 kDa and 126 kDa protein genes, while the 30 kDa and 17.5 kDa protein genes showed relatively little change between ToMV isolates. Previous research has shown that the 130 kDa and 180 kDa proteins are involved in viral RNA replication, whereas the 30-kDa protein and CP are dispensable for replication. The 130 kDa protein contains a methyltransferase-like domain that functions in RNA capping and an RNA helicase-like domain. The read-through region of the 180 kDa protein contains an RNA polymerase-like domain. These three domains are conserved among the replication proteins of the members of the alphavirus-like superfamily [28]. Across the tomato brown rugose fruit virus (ToBRFV) genome, the CP gene accumulated the least number of mutations. It showed high conservation, as evidenced by the lowest haplotype and nucleotide diversity among all genes [29]. The studies in ToMV showed that the CP gene is highly conserved to preserve elicitor recognition by the N’ gene in tobacco.

In contrast, the highest nucleotide diversity was observed for the MP gene. Recent studies showed the MP as an essential protein for overcoming resistance in plants containing the Tm-22 gene [30]. Several non-synonymous mutations were seen in the MP gene; nevertheless, none were critical amino acid changes, except for one isolate with a single amino acid change in this study. The MP appears to be under negative selection pressure, as any mutations in the critical amino acid positions are lethal to the virus. Negative selection pressure has been commonly found in other tobamoviruses, such as tobacco mosaic virus, tobacco mild green mosaic virus, ToBRFV, and pepper mild mottle virus [29]. Hu et al. (2012) suggested that the ToMV CP and the 3‘ noncoding region might play roles in determining host specificity and symptom induction. We used all current GenBank CP sequences to construct a phylogenetic tree, which showed that although there was a distinguished grouping with all Asian strains, the samples collected from three northern Chinese provinces clustered into the same large lateral branch without a distinguished grouping, This result was consistent with the results reported in Iran [18]. Furthermore, a single nucleotide polymorphism analysis of the complete genome revealed that the largest variation was in the 5′ UTR, while that of the CP gene was small and conservative. CP in tobamoviruses has been reported to be very stable and is considered to have a role in host adaptation [31].

Unlike synonymous mutations, non-synonymous mutations are susceptible to natural selection. Therefore, it is important to understand the ratio of synonymous and non-synonymous mutations expressed in dN/dS when performing an evolutionary analysis, with dN/dS < 1 indicating purification selection. Our results showed that the dN/dS values of the strains from the four sampled regions were all less than 1, suggesting that the ToMV populations were under purification selection. Although there is a possible distribution of different ToMV strains in China, purification selection was present in general, which could and should be used to establish an efficient control strategy for this virus. Plant-virus interactions have been suggested to play an essential role in the genetic divergence of tobamoviruses. ToMV has the characteristics of large population size, high replication rate, lack of collation activity of complex enzymes and small genome, and the genome is prone to mutation, thus enhancing the adaptability of the virus population. The wide range of ToMV hosts and complex ecological conditions, irrigation and other agricultural activities will promote the occurrence of diseases. Therefore, ToMV is very likely to break out in local areas, and timely monitoring of gene variation dynamics of ToMV is of great significance for disease prevention and control.

## Figures and Tables

**Table 1:** Primers used for the detection of tomato mosaic virus (ToMV) in tomato plants.

Primers	Sequence (5′-3′)	Temperature (°C)	Position (bp)	Reference
Primers used for virus detection				
ToMV-f	CAAATCCTCAAAAAGAGGTCCG	50	5540–5561	This study
ToMV-r	CAAACTTTATATTTCAGCACC		6189–6209	
Primers used for full-length cDNA amplification				
ToMV-1F	GCCGTATTTTTACAACAATTACCAAC	56	1–23	This study
ToMV-1R	GGGGCTACCAGGCTGTCTATAAAGT		2216–1193	
ToMV-2F	CAACAGCTAGTTCGTTAATTCATAA	53	2116–2140	This study
ToMV-2R	TTACCGATAAAAGTTGTAACATCACCAC		4432–4404	
ToMV-3F	CAATGGACGTACTTGAGTTGGATG	55	4207–4230	This study
ToMV-3R	TTATATATGGGCCCCAACCGG		6383–6369	
Primers used for 5′/3′ RACE				
TO5SP1	AAGTCAACCTGTCTCCATC	-	850–831	[12]
TO5SP2	GCTTTGGTTGCAATAAGCGTCTG	-	265–243	
TO3SP1	GTATGGGCTGACCCTATAGA	-	5751–5770	[12]
TO3SP2	TTGAAAGTATGTCTGGGTTG	-	6136–6156	

**Table 2:** PCR detection of tomato mosaic virus (ToMV) in tomato leaf samples in China.

Province	Location	No. of Leaf Sample Tested	No. of ToMV-Positive Samples	ToMV Sample Detection Rate (%)
Shandong	Liaocheng	54	30	55.56
	Shouguang	87	74	85.06
	Taian	73	58	79.45
	Zibo	90	15	16.67
	Jining	32	11	34.38
	Yantai	61	50	81.97
	Dezhou	9	6	66.67
Inner Mongolia	Hohhot	6	6	100.00
Shanxi	Jinzhong	13	11	84.62
Total	-	425	261	61.41

**Table 3:** Tomato mosaic virus mutation rate of the complete genome (%).

Genomic Region	Nucleotide Number	Nucleotide Mutation	Nucleotide Mutation Rate%	Amino Acid Number	Amino Acid Mutation	Amino Acid Mutation Rate%
5′UTR	71	4	5.6	23	-	-
126 KDa	3351	64	1.9	1117	7	0.6
183 KDa	4851	93	1.9	1617	13	0.8
MP	795	12	1.5	265	0	0
CP	480	12	2.5	160	1	0.6
3′UTR	201	6	2.9	67	-	-

**Table 4:** Genetic parameters, neutrality test, and selection pressure on tomato mosaic virus subpopulations based on geographic origin.

	No. of Sequences ^a^	Nucleotides ^b^	h ^c^	Hd ^d^	S ^e^	Eta ^f^	π ^g^	dN/dS	Tajima’s D	Fu & Li’s D	Fu & Li’s F
A	89	480	52	0.973 (±6 × 10^−5^)	185	212	0.040	0.066	−1.848 *	−3.689 *	−3.490 *
B	39	480	23	0.950 (±4 × 10^−4^)	39	40	0.010	0.138	−1.825 *	−1.775 **	−2.126 **
C	13	480	9	0.936 (±2.57 × 10^−3^)	18	18	0.008	0.135	−1.411 **	−1.153 **	−1.396 **
D	4	480	4	1.000 (±3.125 × 10^−2^)	75	76	0.080	0.055	−0.828 **	−0.786 **	−0.848 **

^a^ The number of ToMV complete CP sequences, ^b^ The nucleotides of CP Protein, ^c^ Haplotype number, ^d^ Haplotype diversity, ^e^ Number of polymorphic (segregating) sites, ^f^ Total number of mutations, ^g^ Nucleotide diversity * *p* < 0.05; ** *p* < 0.01. A (Asia), B (Europe), C (Africa) and D (America).

## Data Availability

All data used in this study are already provided in the manuscript in its required section. There are no underlying data available.

## Supplementary Materials

The following supporting information can be downloaded at: https://www.mdpi.com/article/10.3390/v15071617/s1, Figure S1: Nucleotide sequence mismatch distribution of ToMV isolates from different regions A (Asia), B (Europe), C (Africa) and D (America); Table S1: Information for tomato mosaic virus (ToMV) isolates used in this study; Table S2: Identities of full genome sequences between GS100 isolate and others in GenBank/%; Table S3: Identity of nucleotide sequences in this study; Table S4: Analysis of coat protein gene of ToMV in four regions; Table S5: Analysis of coat protein gene of ToMV in six provinces of China.

## References

[1] Ghorbani A., Razavi S.M., Ghasemi Omran V.O., Pirdashti H. (2018). Piriformospora indica inoculation alleviates the adverse effect of NaCl stress on growth, gas exchange and chlorophyll fluorescence in tomato (*Solanum lycopersicum* L.). Plant Biol..

[2] Desneux N., Luna M.G., Guillemaud T., Urbaneja A. (2011). The invasive South American tomato pinworm, Tuta absoluta, continues to spread in Afro-Eurasia and beyond: The new threat to tomato world production. J. Pest Sci..

[3] Prasad A., Sharma N., Hari-Gowthem G., Muthamilarasan M., Prasad M. (2020). Tomato yellow leaf curl virus: Impact, challenges, and management. Trends Plant Sci..

[4] Fukuhara T., Tabara M., Koiwa H., Takahashi H. (2020). Effect of asymptomatic infection with southern tomato virus on tomato plants. Arch. Virol..

[5] Fiallo-Olivé E., Navas-Castillo J. (2019). Tomato chlorosis virus, an emergent plant virus still expanding its geographical and host ranges. Mol. Plant Pathol..

[6] Yan Z.Y., Ma H.Y., Han S.L., Geng C., Tian Y.P., Li X.D. (2019). First report of tomato brown rugose fruit virus infecting tomato in China. Plant Dis..

[7] Kumar S., Udaya Shankar A.C., Nayaka S.C., Lund O.S., Prakash H.S. (2011). Detection of tobacco mosaic virus and tomato mosaic virus in pepper and tomato by multiplex RT–PCR. Lett. Appl. Microbiol..

[8] FAO (2018). Statistical Database of the Food and Agricultural Organization of the United Nations: FAOSTAT. http://www.fao.org/faostat/en/#data.

[9] Mrkvová M., Hančinský R., Grešíková S., Kaňuková Š., Barilla J., Glasa M., Hauptvogel P., Kraic J., Mihálik D. (2022). Evaluation of new polyclonal antibody developed for serological diagnostics of tomato mosaic virus. Viruses.

[10] Rangel E.A., Alfaro-Fernández A., Font-San-Ambrosio M.I., Luis-Arteaga M., Rubio L. (2011). Genetic variability and evolutionary analyses of the coat protein gene of tomato mosaic virus. Virus Genes.

[11] Gibbs A. (1999). Evolution and origins of tobamoviruses. Philos. Trans. R. Soc. Lond. Ser. B Biol. Sci..

[12] Hu Q., Jiang T., Xue C., Zhou X. (2012). Characterization and complete nucleotide sequence of two isolates of tomato mosaic virus. J. Phytopathol..

[13] Pozharskiy A., Kostyukova V., Taskuzhina A., Nizamdinova G., Kisselyova N., Kalendar R., Karimov N., Gritsenko D. (2022). Screening a collection of local and foreign varieties of *Solanum lycopersicum* L. in Kazakhstan for genetic markers of resistance against three tomato viruses. Heliyon.

[14] Diaz-Lara A., Santamaria L., Martin R.R. (2017). Identification of tomato mosaic virus (ToMV) and potato latent virus (PotLV) as mixed infection in Chinese lantern (*Physalis alkekengi*) expressing virus-like disease symptoms. Plant Dis..

[15] Pagán I., Firth C., Holmes E.C. (2010). Phylogenetic analysis reveals rapid evolutionary dynamics in the plant RNA virus genus tobamovirus. J. Mol. Evol..

[16] Xu Y., Zhang S., Shen J., Wu Z., Du Z., Gao F. (2021). The phylogeographic history of tomato mosaic virus in Eurasia. Virology.

[17] Fujisaki K., Ishikawa M. (2008). Identification of an Arabidopsis thaliana protein that binds to tomato mosaic virus genomic RNA and inhibits its multiplication. Virology.

[18] Aghamohammadi V., Rakhshandehroo F., Shamsbakhsh M., Palukaitis P. (2013). Distribution and genetic diversity of tomato mosaic virus isolates in Iran. J. Plant Pathol..

[19] Kumar S., Stecher G., Tamura K. (2016). MEGA7: Molecular evolutionary genetics analysis version 7.0 for bigger datasets. Mol. Biol. Evol..

[20] Rozas J., Ferrer-Mata A., Sánchez-DelBarrio J.C., Guirao-Rico S., Librado P., Ramos-Onsins S.E., Sanchez-Gracia A. (2017). DnaSP 6: DNA Sequence Polymorphism Analysis of Large Data Sets. Mol. Biol. Evol..

[21] Martin D.P., Murrell B., Golden M., Khoosal A., Muhire B. (2015). RDP4: Detection and analysis of recombination patterns in virus genomes. Virus Evol..

[22] Ullah N., Akhtar K.P., Saleem M.Y., Habib M. (2019). Characterization of tomato mosaic virus and search for its resistance in Solanum species. Eur. J. Plant Pathol..

[23] Ge B.B., Liu G.J., Wang H.Q. (2012). First report of tomato mosaic virus infecting pepino in China. Plant Dis..

[24] Hashemi S.S., Rakhshandehroo F., Shahraeen N. (2014). First report of tomato mosaic virus on common sow thistle in Iran. Plant Dis..

[25] Fillmer K., Adkins S., Pongam P., D’Elia T. (2015). Complete genome sequence of tomato mosaic virus isolated from jasmine in the United States. Genome Announc..

[26] Broadbent L. (1976). Epidemiology and control of tomato mosaic virus. Annu. Rev. Phytopathol..

[27] Fraile A., García-Arenal F. (2010). The coevolution of plants and viruses: Resistance and pathogenicity. Adv. Virus Res..

[28] Lewandowski D.J., Dawson W.O. (2000). Functions of the 126-and 183-kDa proteins of tobacco mosaic virus. Virology.

[29] Abrahamian P., Cai W., Nunziata S.O., Ling K.-S., Jaiswal N., Mavrodieva V.A., Rivera Y., Nakhla M.K. (2022). Comparative analysis of tomato brown rugose fruit virus isolates shows limited genetic diversity. Viruses.

[30] Hak H., Spiegelman Z. (2021). The Tomato brown rugose fruit virus movement protein overcomes Tm-22 resistance in tomato while attenuating viral transport. Mol. Plant-Microbe Interact..

[31] Dombrovsky A., Smith E. (2017). Seed transmission of Tobamoviruses: Aspects of global disease distribution. Adv. Seed Biol..

