# Supplementary material for: Genetic Variability and Molecular Evolution of Tomato Mosaic Virus Populations in Three Northern China Provinces
Source: Viruses. 2023 Jul 24;15(7):1617. doi: 10.3390/v15071617 (PMC10383530; doi:10.3390/v15071617)
Supplement: Supplementary file 1 [file viruses-15-01617-s001.zip › viruses-2448380-suppl.pdf]

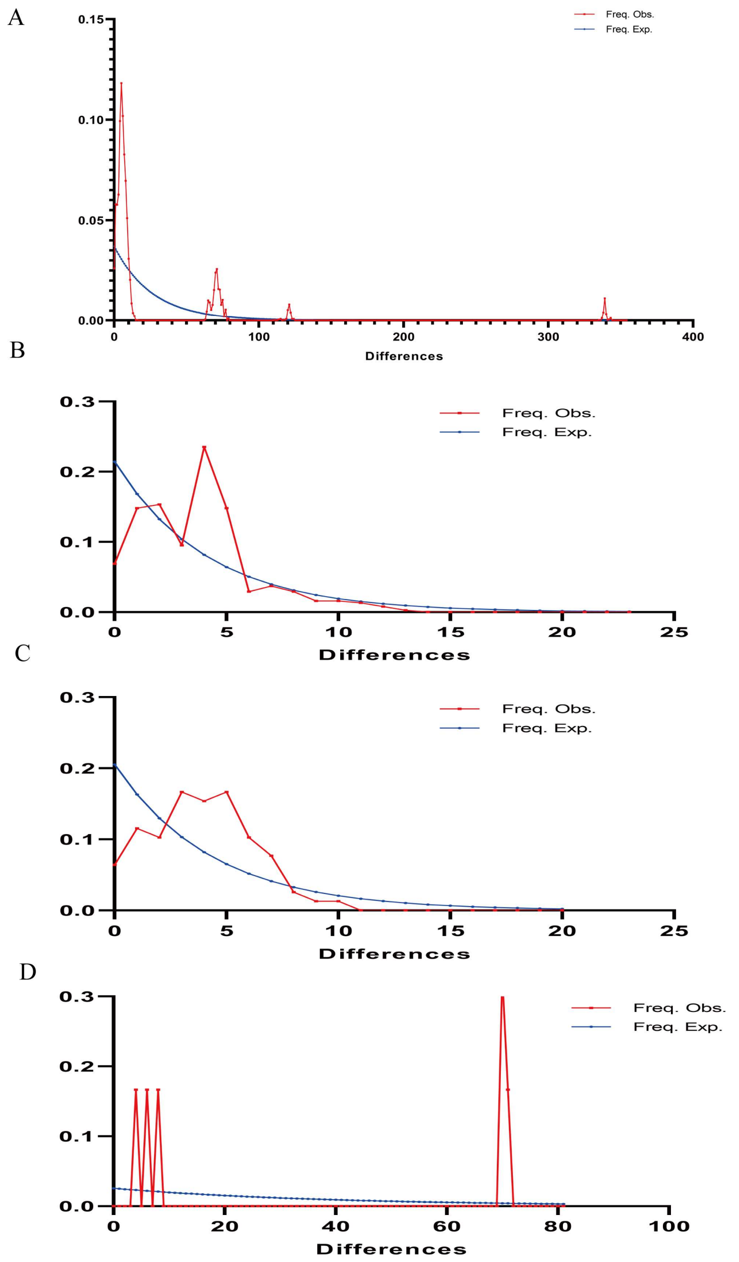

Figure S1. Nucleotide sequence mismatch distribution of ToMV isolates from different regions A (Asia), B (Europe), C (Africa) and D (America).

Table S1. Information for tomato mosaic virus (ToMV) isolates used in this study.

	GenBank acc. No.	Isolate	Genome	Host	Origin
Asia (A)	KY949475	GS102	CP/complete sequence	Tomato	China
	KY949480	Neimenggu	CP/ complete sequence	Solanum lycopersicum	China
	KY949481	LC	CP/ complete sequence	Solanum lycopersicum	China
	KY967219	SX	CP/ complete sequence	Lycopersicon esculentum	China
	KY967220	SGHG	CP/ complete sequence	Lycopersicon esculentum	China
	KY967221	SGZZ	CP/ complete sequence	Lycopersicon esculentum	China
	KY967222	Tai'an	CP/complete sequence	Lycopersicon esculentum	China
	KY967223	ZB	CP/ complete sequence	Lycopersicon esculentum	China
	KY967224	JN	CP/ complete sequence	Lycopersicon esculentum	China
	KY967225	LY	CP/ complete sequence	Lycopersicon esculentum	China
	KY967226	HY	CP/ complete sequence	Lycopersicon esculentum	China
	KY967227	YT	CP/ complete sequence	Lycopersicon esculentum	China

**KY967228**	**HHHT**	**CP/complete sequence**	**Lycopersicon esculentum**	**China**
**MF002479**	**HHHT4**	**CP/ complete sequence**	**Lycopersicon esculentum**	**China**
**MF002480**	**HHHT3**	**CP/ complete sequence**	**Lycopersicon esculentum**	**China**
**MF002481**	**HHHT2**	**CP/ complete sequence**	**Lycopersicon esculentum**	**China**
**MF002482**	**HHHT1**	**CP/ complete sequence**	**Lycopersicon esculentum**	**China**
**MF002483**	**SX1**	**CP/ complete sequence**	**Lycopersicon esculentum**	**China**
**MF002484**	**GS100**	**CP/complete sequence**	**Lycopersicon esculentum**	**China**
**MF002485**	**GS1**	**CP/ complete sequence**	**Lycopersicon esculentum**	**China**
**MF002486**	**SX_SG2**	**CP/ complete sequence**	**Lycopersicon esculentum**	**China**
**MF002487**	**Tai,an1**	**CP/ complete sequence**	**Lycopersicon esculentum**	**China**
**MF002488**	**Tai,an2**	**CP/ complete sequence**	**Lycopersicon esculentum**	**China**
**MF002489**	**LC1**	**CP/ complete sequence**	**Lycopersicon esculentum**	**China**
**MF002490**	**JX**	**CP/complete sequence**	**Lycopersicon esculentum**	**China**
**MF002491**	**XT**	**CP/ complete sequence**	**Lycopersicon esculentum**	**China**
AF155507	K	CP/ complete sequence	tomato	China
AJ132845	S-1	CP/ complete sequence	tomato	China
AJ417701	TL	CP/ complete sequence	camellia	China
FN985165	XJT-1	CP/complete sequence	tomato	China
GQ280794	N5	CP/ complete sequence	tomato	China
KJ207374	Penghu	CP/ complete sequence	Pepino	China
**KY652976**	**Beijing**	**CP**	**tomato**	**China**
**KY652979**	**Shandong**	**CP**	**tomato**	**China**
**KY629780**	**Shanxi**	**CP**	**tomato**	**China: Shanxi**
**KY629781**	**Neimenggu**	**CP**	**tomato**	**China: Neimenggu**
**KY629782**	**Shouguang**	**CP**	**tomato**	**China: Shouguang**
**KY629783**	**Liaocheng**	**CP**	**tomato**	**China: Liaocheng**
**KY629784**	**Tai'an**	**CP**	**tomato**	**China: Tai'an**
Z98201	S1	CP	-	China
JQ085379	Penghu	CP	pepino	China: Taiwan
AY313136	H. rosa-sinensis	CP	H. rosa-sinensis	China
JX857634	LZ2	CP	Medicago sativa	China
AY383730	Lisianthus	CP	lisianthus	China: Taiwan
JX025562	P1	CP	Solanum muricatum	China
JX025563	P2	CP	Solanum muricatum	China
JX025564	T1	CP	Lycopersicon esculentum	China
JX025565	G1	CP	Gomphrena globosa	China
JX025566	G2	CP	Gomphrena globosa	China
JQ966553	HX-T2	CP	Solanum lycopersicum	China
JQ966550	HX-T5	CP	Capsicum annuum	China
JQ966551	HX-T4	CP	Capsicum annuum	China
JQ966552	HX-T3	CP	Capsicum annuum	China
JQ954826	HX-T1	CP	tomato	China
JX982095	RS8	CP	pepino	China
JX982097	RS1	CP	pepino	China
KY977427	ToMV-X1-3	CP	river water	China
AB083196	L11A-Fukushima	CP/ complete sequence	tomato	Japan
AB355139	L11Y	CP/ complete sequence	tomato	Japan
X02144	OM	CP/ complete sequence	tomato	Japan
MH507165	GW1	CP/ complete sequence	Solanum lycopersicum	South Korea
MH507166	GW2	CP/complete sequence	Solanum lycopersicum	South Korea
AF260730	potato1	CP	Solanum tuberosum	Korea
HM623426	Ls-K	CP	Lycopersicon esculentum	South Korea
EU885417	ToMV-tom	CP	tomato	South Korea
MG345100	BK1	CP	Solanum lycopersicum	South Korea
AJ243571	K1	CP/ complete sequence	tobacco	Kazakhstan
Z92909	K2	CP/ complete sequence	tobacco	Kazakhstan
KX424990	SN SLM 1	CP	Solanum nigrum	India
JX121576	Ham-Tom-6	CP	tomato	Iran
JX121574	Ker.Pep.3	CP	pepper	Iran

	JX112024	Ham.Tom.9	CP	tomato	Iran
	JX121575	Ker.Tom.41	CP	tomato	Iran
	JX121573	Ker.Pep.38	CP	pepper	Iran
	JX121572	Ker.Pep.18	CP	pepper	Iran
	JX112025	Ham.Tom.19	CP	tomato	Iran
	JX121570	Ker.Pha.2	CP	kidney bean	Iran
	JX121571	Ker.Dat.24	CP	thorn apple	Iran
	JX135609	Jir.Che.4	CP	common lambsquarters	Iran
	KC914397	Sm-T107	CP	Solanum lycopersicum	Iran
	KC914398	Sm-E59	CP	Solanum melongena	Iran
	KC914399	Kh-P100	CP	Solanum tuberosum	Iran
	KC914400	Kh-C239	CP	Cucumis sativus	Iran
	HQ593624	G6	CP	eggplant	Iran
	HQ593625	Q15	CP	tomato	Iran
	HQ593626	T16	CP	tomato	Iran
	HQ593627	G26	CP	tomato	Iran
	HQ593616	TMV-Jahrum	CP	Chenopodium murale	Iran
Europe (B)	AY063743	S15	CP	tomato	Spain
	JF810425	S1	CP	tomato	Spain
	JF810426	S2	CP	tomato	Spain
	JF810427	S5	CP	tomato	Spain
	JF810428	S17	CP	tomato	Spain
	JF810429	S18	CP	tomato	Spain
	JF810430	S19	CP	tomato	Spain
	JF810431	S20	CP	tomato	Spain
	JF810432	S21	CP	tomato	Spain
	JF810433	S22	CP	tomato	Spain
	JF810434	S9	CP	tomato	Spain
	JF810435	S10	CP	tomato	Spain
	JF810436	S16	CP	tomato	Spain
	JF810437	S14	CP	tomato	Spain
	JF810438	S15	CP	tomato	Spain
	JF810439	S25	CP	tomato	Spain
	JN381939	T11	CP	tomato	Spain
	JN381943	T15	CP	tomato	Spain
	JN381944	T16	CP	tomato	Spain
	JN381938	T10	CP	tomato	Spain
	JN381940	T12	CP	tomato	Spain
	JN381941	T13	CP	tomato	Spain
	JN381942	T14	CP	tomato	Spain
	JN381931	T1	CP	tomato	Spain
	JN381932	T2	CP	tomato	Spain
	JN381933	T3	CP	tomato	Spain
	JN381934	T4	CP	tomato	Spain
	JN381935	T5	CP	pepper	Spain
	JN381936	T8	CP	pepper	Spain
	JN381937	T9	CP	tomato	Spain
	KY810788	FERA_160205	CP/ complete sequence	Solanum lycopersicum	United Kingdom
	DQ873692	ToMV1-2	CP/complete sequence	tomato	Germany
	AJ429084	DSMZ PV-0135	CP	Capsicum annum L.	Germany
	AJ429085	DSMZ PV-127	CP	Capsicum annum L.	Germany
	AJ429086	DSMZ PV-472	CP	Capsicum annum L.	Germany
	AJ429083	437	CP	Capsicum annum L.	Germany
	MG018614	105-12	CP	tomato	Serbia
	KP861861	ukr3	CP	tomato	Ukraine
	KY912162	SL-1	CP/ complete sequence	tomato	Slovakia
Africa (C)	LN827930	AH1	CP	tomato	Egypt
	LN827931	AH2	CP	tomato	Egypt

	LN827932	AH3	CP	tomato	Egypt
	LN827933	AH4	CP	tomato	Egypt
	LN827934	AH5	CP	tomato	Egypt
	LN827935	AH6	CP	tomato	Egypt
	LN827936	AH7	CP	tomato	Egypt
	LN827937	AH8	CP	tomato	Egypt
	LN827938	AH9	CP	tomato	Egypt
	LN827939	AH10	CP	-	Egypt
	KU321698	AH4	CP/ complete sequence	tomato	Egypt
	KX711903	mutoko	CP/ complete sequence	Solanum lycopersicum	Zimbabwe
	MG456601	ToMV-Ug	CP/ complete sequence	Solanum lycopersicum	Uganda
	MH006893	Qua+1	CP	Solanum lycopersicum	Brazil
America	AF411922	ToMV-SP	CP	tomato	Brazil
(D)	KT923127	-	CP	Nicotiana tabacum	Brazil
	KR537870	99-1	CP/ complete sequence	Jasminum multiflorum	USA
Oceania	AF332868	Queensland	CP/ complete sequence	Lycopersicon esculentum	Australia
-	NC_001367		Tobacco mosaic virus		

Table S2. Identities of full genome sequences between GS100 isolate and others in GenBank /%.

Isolate	GenBank No.	Origin	Host	Genome	5'UTR	184 KDa	126 KDa	MP	CP	3'UTR
GS1	MF002485	China (Shouguang)	Lycopersicon esculentum	99.6	100	99.6	99.3	99.4	99.4	100
SGZZ	KY967221	China (Shouguang)	Lycopersicon esculentum	98.9	100	98.9	99.4	99.0	98.8	98.0
SGHG	KY967220	China (Shouguang)	Lycopersicon esculentum	99.6	100	99.7	99.2	99.5	99.2	99.5
Taian	KY967222	China (Taian)	Lycopersicon esculentum	99.6	100	99.7	99.5	99.4	99.8	99.5
Taian1	MF002487	China (Taian)	Lycopersicon esculentum	99.6	100	99.9	99.8	99.5	99.5	99.5
Taian2	MF002488	China (Taian)	Lycopersicon esculentum	99.2	98.6	99.0	99.0	99.5	99.2	99.4
XT	MF002491	China (Taian)	Lycopersicon esculentum	99.6	100	99.7	99.5	99.4	99.8	99.5
LC1	MF002489	China (Liaocheng)	Lycopersicon esculentum	99.6	100	99.7	99.3	99.5	99.2	98.5
JX	MF002490	China (Jinxiang)	Lycopersicon esculentum	98.9	100	98.9	99.4	99.1	98.8	98.0
ZB	KY967223	China (Zibo)	Lycopersicon esculentum	98.8	100	98.9	99.3	99.0	98.8	99.0
JN	KY967224	China (Jinan)	Lycopersicon esculentum	98.9	100	98.8	99.2	99.0	98.8	99.0
LY	KY967225	China (Laiyang)	Lycopersicon esculentum	98.9	100	98.8	99.1	99.0	98.8	99.0
HY	KY967226	China (Haiyang)	Lycopersicon esculentum	98.9	100	98.8	99.0	99.0	98.9	98.0
YT	KY967227	China (Yantai)	Lycopersicon esculentum	98.9	97.3	98.9	99.2	99.1	98.8	98.0
SX	KY967219	China (Shanxi)	Lycopersicon esculentum	98.5	98.6	98.7	98.7	99.0	98.1	98.0
SX1	MF002483	China (Jinzhong)	Lycopersicon esculentum	98.6	98.6	98.8	99.2	98.9	98.3	98.0
HHHT	KY967228	China (Neimenggu)	Lycopersicon esculentum	98.7	98.6	98.8	99.1	99.0	98.1	97.0
HHHT1	MF002482	China (Huhehaote)	Lycopersicon esculentum	98.6	98.6	98.7	98.9	98.2	98.1	98.5
HHHT2	MF002481	China (Huhehaote)	Lycopersicon esculentum	98.7	98.6	98.8	99.2	98.6	98.1	98.5

HHHT3	MF002480	China (Huhehaote)	Lycopersicon esculentum	98.7	98.6	98.7	98.7	98.9	98.3	98.5
HHHT4	MF002479	China (Huhehaote)	Lycopersicon esculentum	98.7	98.6	98.8	98.9	98.9	98.3	98.0
L11A-Fukushima	AB083196	Japan	tomato	99.1	97.3	96.9	99.0	99.1	98.8	98.5
L11Y	AB355139	Japan	Tobacco	96.3	97.3	97.0	99.1	99.1	98.3	98.5
K	AF155507	China	tomato	98.9	97.3	96.7	98.7	98.6	98.8	98.5
Queensland	AF332868	Australia	tomato	99.2	97.3	98.0	99.0	99.1	99.0	98.0
ToMV1-2	DQ873692	Germany	tomato	99.3	97.3	97.2	99.3	99.1	99.2	98.5
N5	GQ280794	China	Lycopersicon esculentum	98.0	97.3	96.8	98.8	99.0	98.1	97.0
Penghu	KJ207374	Taiwan	Solanummuricatum	98.8	97.3	96.8	99.0	99.1	98.1	96.1
AH4	KU321698	Egypt	tomato	93.8	97.3	96.5	96.6	98.6	98.3	96.6
OM	X02144	OM	tomato	99.2	97.3	97.1	99.1	99.1	98.8	98.5
K2	Z92909	Kazakhstan	tomato	90.1	97.3	96.9	99.0	99.2	97.7	97.5
99-1	KR537870	USA	Jasminum multiflorum	98.6	100	99.0	99.0	99.3	99.0	93.2
TL	AJ417701	China	camellia	73.2	100	99.1	99.0	99.2	99.2	99.6
FERA_160205	KY810788	United Kingdom	Solanum lycopersicum	98.9	92.1	99.2	99.2	97.5	99.0	98.1
GS102	KY949475	China (Shouguang)	Lycopersicon esculentum	76.9	100	90.6	93.5	100	100	100
GW1	MH507165	South Korea	Solanum lycopersicum	99.0	100	99.2	99.3	99.2	99.2	99.2
GW2	MH507166	South Korea	Solanum lycopersicum	99.0	100	99.2	99.3	99.2	99.2	99.2
K1	AJ243571	Kazakhstan	tobacco	73.3	100	99.3	99.3	99.4	98.2	98.9
LC	KY949481	China (Liaocheng)	Lycopersicon esculentum	99.6	100	99.8	99.8	99.6	99.3	99.6
mutoko	KX711903	Zimbabwe	Solanum lycopersicum	98.7	100	99.1	99.2	98.9	98.9	94.3
Neimenggu	KY949480	China (Neimenggu)	Lycopersicon esculentum	98.8	98.0	99.1	99.2	99.2	98.5	98.5
S-1	AJ132845	China	tomato	98.9	100	99.0	99.0	99.3	99.3	95.5
SL-1	KY912162	Slovakia	tomato	98.8	100	99.2	99.2	98.9	97.9	99.2
SX2	MF002486	China (Weifang)	Lycopersicon esculentum	99.6	100	99.8	99.8	99.6	99.3	99.6
ToMV-Ug	MG456601	Uganda	Solanum lycopersicum	98.8	100	99.0	99.1	99.3	99.0	98.5
XJT-1	FN985165	China	tomato	98.9	100	99.1	99.1	99.0	99.2	98.9

Table S4. Analysis of coat protein gene of ToMV in four regions.

Region	Region	|Fst|
A	B	0.04543
A	C	0.13702
A	D	0.09459
B	C	0.38170
B	D	0.00734
C	D	0.07137

A (Asia), B (Europe), C (Africa) and D (America).

Table S5. Analysis of coat protein gene of ToMV in six provinces of China.

Region	Region	|Fst|
a	b	0.34000
a	c	0.50413
a	d	0.09002
a	e	0.12950
a	f	0.13067
b	c	0.18333

b	d	0.05226
b	e	0.35294
b	f	0.20472
c	d	0.13004
c	e	0.51145
c	f	0.35632
d	e	0.05694
d	f	0.05290
e	f	0.07843

a (Shandong), b (Shanxi), c (Inner Mongolia), d (Beijing), e (Zhejiang), f (Gansu).
